# Simulated nonlinear genetic and environmental dynamics of complex traits

**DOI:** 10.1017/S0954579421001796

**Published:** 2022-03-03

**Authors:** Michael D. Hunter, Kevin L. McKee, Eric Turkheimer

**Affiliations:** 1 Department of Human Development and Family Studies, The Pennsylvania State University, University Park, PA, USA; 2 Center for Neuroscience, University of California, Davis, Davis, CA, USA; 3 Department of Psychology, University of Virginia, Charlottesville, VA, USA

**Keywords:** behavior genetics, dynamical systems, GWAS, heritability, simulation

## Abstract

Genetic studies of complex traits often show disparities in estimated heritability depending on the method used, whether by genomic associations or twin and family studies. We present a simulation of individual genomes with dynamic environmental conditions to consider how linear and nonlinear effects, gene-by-environment interactions, and gene-by-environment correlations may work together to govern the long-term development of complex traits and affect estimates of heritability from common methods. Our simulation studies demonstrate that the genetic effects estimated by genome wide association studies in unrelated individuals are inadequate to characterize gene-by-environment interaction, while including related individuals in genome-wide complex trait analysis (GCTA) allows gene-by-environment interactions to be recovered in the heritability. These theoretical findings provide an explanation for the “missing heritability” problem and bridge the conceptual gap between the most common findings of GCTA and twin studies. Future studies may use the simulation model to test hypotheses about phenotypic complexity either in an exploratory way or by replicating well-established observations of specific phenotypes.

## Introduction

Although it has been clear for one hundred years that differences in genotype are correlated with differences in complex human behavior (e.g., Galton, 1869; Fisher, 1918), the biological mechanisms underlying such correlations have proven to be very difficult to specify. Generally, the proportion of phenotypic variability attributable to genetic sources is known as heritability. The heritability of behavior was originally detected well-before the discovery of DNA, on the basis of quantitative genetic analysis of variation in genetic relatedness among family members, especially identical and fraternal twins. Under the classical twin model, similarities of identical and fraternal twins can be used to partition the variance in a phenotype into three components: the additive effects of genes (A), the environmental effects of families that make siblings raised together more similar (C, the common or shared environment), and environmental effects that make children raised in the same family more different (E, usually called the nonshared environment). Other variance components are possible as well, such as the total additional genetic effect due to allelic dominance (D). The A component captures the additive effects of alleles at each genetic locus, known as the narrow-sense heritability, whereas the total effects of all genes compose the broad sense heritability. Often, the sum of the A and D components approximates the broad sense heritability, even though it does not capture all possible nonlinear gene effects. Both of these heritabilities can, in turn, be modified by environmental activation and suppression of genetic effects. This paper focuses on the distinction between true, underlying genetic effects as they involve environmental interactions and the narrow sense heritability estimated by various procedures.

A second theme of this paper is the role that nonlinearity and nonadditivity play in estimates of heritability. In the presence of gene–gene interactions and gene-by-environment interactions, the conventional notion of heritability loses its focus. Plomin et al., (1977, p. 311, Equation 1) explained gene-by-environment interactions as akin to regression interaction coefficients: the effect of the genes depends on the level of the environment. Just as with conventional regression models, it is possible to have a significant interaction effect in the absence of a main effect. Such a result implies that the effect of one factor is entirely dependent on the presence of the other. For example, the heritability of addiction to a substance requires that use of the substance was at some point initiated, an environmental exposure. However, the notion of heritability can be preserved by allowing the heritability to be a nonlinear function of the environment (e.g., Turkheimer et al., 2003, p. 626, Equation 3). The same argument applies to gene–gene interaction. Lykken and colleagues (Lykken, 1982; Lykken, et al., 1992) described a set of phenotypes that appeared to be genetically determined and yet did not run in families. Lykken argued that these traits were driven by interactions between large numbers of genes (e.g., 15-way gene-gene interactions). Using the regression analogy, the gene-gene interactions were obfuscating the linear gene “main effects” such that identical twins matched on traits whereas fraternal twins were no more similar than unrelated individuals.

Broadly, pairs of identical twins are more similar than pairs of fraternal twins for almost every behavioral phenotype that has ever been studied (Polderman et al., 2015). More generally, phenotypic similarity increases as a monotonic function of genotypic similarity for nearly all phenotypes, ranging from genetically unrelated adopted children to genetically identical monozygotic twins. Moreover, twin and family analyses have generally suggested that the common environmental component of families (C) has little or no effect. The remarkable regularity of results of twin and family studies has been summarized under the “Three Laws of Behavior Genetics” (Turkheimer, 2000). However, in spite of this regularity, an important complication arises related to the role of common and unique environments. Studies of some behavioral phenotypes have estimated the phenotypic correlation among monozygotic (MZ) twins to be more than twice that for dizygotic (DZ) twins. In the ACE model, this can result in a negative value of C, and thus suggests that an additive, linear common environment is not an adequate explanation. In fact, Giangrande and Turkheimer (2019) reported that 56%s of their 1,300 reviewed studies implied negative values of C. So, there is reason to suppose that the environment and gene-environment interactions may not be well-captured by standard methods in behavior genetics such as the ACE model under the classical twin design.

The causal role of the environment in the development of behavior is, if anything, even more intractable than that of genetics. Some behavior geneticists have expressed doubt about the very existence of systematic environmental effects that apply equally to all people (e.g., Plomin, 2019). In twin and adoption studies, simple models yield replicable findings of additive genetic variance without specifying biological mechanisms of the genes, yet similar models of the shared environment often find no shared environmental effects at all (i.e., the C component is estimated near zero). In a classic paper, Plomin and Daniels (1987) suggested that the absence of C variance, in contrast to large nonshared environmental (E) components, meant that environmental causes of phenotypic differences operated mostly within families, as opposed to the familiar sociological candidates for environmental effects, like socioeconomic status, which vary almost entirely between families.^1^ However, even though twin studies estimate substantial non-shared environmental variance components, these latent components cannot generally be decomposed into detectable causal effects of the actual environmental events they comprise. When Turkheimer and Waldron (2000) performed a meta-analysis of measured within-family environmental differences among siblings, they found that even though the nonshared environmental variance component was substantial, the individual effects of measured within-family environmental variables were negligible.

As a means of understanding the disconnect between reliable variance components and inscrutable causal processes, Turkheimer and Gottesman (1996) and Turkheimer (2004) developed a simulation of a simple genotype–environment system that had equally strong, causal effects from genes and environmental factors, but only the gene effects could be recovered from phenotypic observations. The simulation was based on gravitational planetary models. A set of fixed “genes” was randomly arranged in a two dimensional space. Also located in the space were an “environment” and a “phenotype.” In order to circumvent empirical debates about the relative strength of genetic versus environmental factors, the total influence of genes and environment was set to be equal. The simulated system proceeded to move according to the following rules:

1. The phenotype is attracted to the genes (additive genetic effects).
2. The phenotype is attracted to the environment (additive environmental effects).
3. The environment is attracted to the phenotype (gene-by-environment correlation).
4. The relative strength of each gene depends on the location of the environment (gene-by-environment interaction).

The final position of the phenotype converged on a location, which was recorded and analyzed in terms of the genotype and the original position of the environment. This simple system reproduced many of the findings of classical twin studies. Twin simulations with identical genotypes produced highly similar outcomes. “DZ” twins – who shared half of their gene locations in the two-dimensional space – showed some similarity but much less than their “MZ” twin counterparts. In general, outcomes were much more predictable from the static genotype than from the dynamic environment. In fact, much of the variability in the simulation was not predictable by either the genes or the environment; the outcome was chaotic and fractal in nature.

In the years since the publication of those simulations, sequencing of the human genome has been completed, and genetic technology based on measured DNA has risen to prominence, to some extent supplanting methods based on genetic relatedness among family members. These technological advances led to an expectation that relatively straightforward biological pathways linking genes to behavior would eventually be discovered (e.g., Plomin & Crabbe, 2000), but such advances have proven much more difficult than anyone expected. Early attempts at gene finding using linkage analysis quickly showed that there were no genes of large effect for behavioral phenotypes (Crow, 2007). Attempts to identify genes of smaller effect via candidate gene association analysis were inconclusive and difficult to replicate (Border et al., 2019). Genome wide association studies (GWAS) have identified single-nucleotide polymorphisms (SNPs) with statistically significant associations with behavioral phenotypes, but at a biological level still face many of the same obstacles as twin and family studies. Although GWAS identifies SNPs that are open to annotation and further analysis, knowledge of statistical associations with SNPs has not yet led to clear cut understanding of biological gene-environment pathways. Moreover, the effects of measured DNA in GWAS do not add up to the heritability that is calculated from twin and family studies, a phenomenon known as missing heritability (Young, 2019).

The most recent DNA-based analyses of the genetics of complex phenotypes, of which human behavior is the ultimate example, have demonstrated that the complexity of developmental processes underlying genetic effects exceeds what might be expected from a straightforward interpretation of the term “polygenic.” That is, nearly all genes might be causally related to a complex developmental process and their effects might not be simple, independent, and additive. Chabris et al., (2015) added a “Fourth Law of Behavior Genetics” to the three that had been proposed for twin genetics (Turkheimer, 2000): “A typical human behavioral trait is associated with very many (i.e., more than can be counted) genetic variants, each of which accounts for a very small percentage of the behavioral variability.” Boyle, et al., (2017) introduced the concept of “omnigenics,” suggesting that cell regulatory networks have the effect of spreading out the effects of causal DNA to the extent that empirical gene-association studies would find that *all* DNA variants may plausibly be associated with complex outcomes. Moreover, causal pathways between genetic variants and phenotypic outcomes are highly complex, largely unknown, and have been shown to be mediated by environmental processes. Genetic associations within sibling pairs (Selzam et al., 2019) or in adoptive families (Cheesman et al., 2020) are reduced compared to estimates between biological families, suggesting that shared environmental characteristics of the family mediate genetic associations. Work by Kong and colleagues (Kong et al., 2018; Young, et al., 2019) has shown that alleles that are not inherited from parents account for a significant portion of the overall correlation between genotype and behavioral phenotypes, a phenomenon they call genetic nurture. Controlling for these processes reduces estimates of SNP heritability and the associated performance of polygenic scores used for genetic prediction (Young et al., 2018; Morris, et al., 2020).

In this paper, we will create a dynamical systems model of the combined phenotype–gene–environment system that extends the previous gravitational planetary model to modern molecular genetic concepts and methods. We next provide greater mathematical detail on the model developed. Subsequently, we use this model to conduct four studies aimed at replicating common findings in modern molecular research on complex phenotypes, and conclude that nonlinear phenotype–gene–environment dynamics can account for our observations. In study 1, we demonstrate the impact of nonlinear gene-by-environment interactions on gene finding with GWAS methods. In study 2, we demonstrate the impact of nonlinear gene-by-environment interactions on the use of polygenic risk scores. In study 3, we demonstrate the impact of nonlinear gene-by-environment interactions on SNP-based estimates of heritability, particularly missing heritability. Finally, in study 4, we show how the degree of relatedness impacts SNP-based estimates of heritability. We conclude by suggesting further questions that can be answered by our dynamical model.

## Model

We model a dynamical system composed of genes, environmental influences, and a phenotype. We simulate genomic vectors, environmental factors, and phenotypic initial conditions, then describe the interdependence between them with a system of ordinary differential equations (e.g., Arnold, 1973; Hirsch, et al., 2003). Genes are static, but have time-varying effects by interacting with the environment. The environment has both static and time-varying components. The phenotype is a result of the genes, their activations, the environment, and its own intrinsic properties.

Core concepts from behavior genetics can be represented in a Newtonian gravitational approach, wherein the mutual influences among genes, environmental factors, and the phenotype are represented by differential equations. These mutual influences are modeled as forces of attraction between the genes, the environmental factors, and the phenotype in the space of possible phenotypic scores. For example, each SNP is represented by a possible phenotypic value toward which the expressed phenotype will move, but the SNP itself does not move: thus, representing the fixed genome and its influence on the phenotype. Similarly, static environmental factors attract the phenotype while remaining fixed in phenotypic space, whereas time-varying environmental factors both pull the phenotype and are pulled by the phenotype. The genes do not have any direct influence on the environmental factors, but rather act upon them indirectly through the phenotype and are activated by the time-varying environmental factors. The magnitudes of the SNP and environmental effects determine the speed at which the phenotype moves toward it and changes over time. The goal of such a model is to quantitatively instantiate the qualitative intuitions gained from empirical research on how genes, environmental factors, and phenotypes interact.

In the model equations to follow, user-controlled simulation parameters are represented by Greek symbols. Table 1 gives the complete list of these parameters with their functions, along with the four dynamic elements of the phenotypic system to be modeled.

The model begins by generating genomes as vectors of allelic values *g_i_* ∈ {0, 1, 2} for each SNP *i* under the standard assumptions of population genetics (i.e., Hardy–Weinberg equilibrium). Thus, *P*(*g_i_* = 0) = *π*^2^, *P*(*g_i_* = 1) = 2*π*(1 − *π*), and *P*(*g_i_* = 2) = (1 − *π)*^2^. For simplicity, the minor allele frequency *π* was the same for all loci. Generating allelic values as independent draws from a categorical distribution has some correspondence to the true underlying biological processes, but lacks other known aspects of the biology. In particular, there is no linkage, linkage disequilibrium, or population structure in the simulated genome.

Next, we use an exponential function to map each SNP by its index *i* to its attractor strength Λ_*i*_. Although Λ_*i*_ is not itself the effect found by regressing the phenotype on SNP_*i*_, it ultimately produces that effect by defining how strongly SNP_*i*_ attracts the phenotype toward a particular point in the phenotype space, as will be defined later.

(1) $${\text{Λ}}_{i}=\exp\left(-\psi \frac{i}{N_{g}}\right),\;i\in 1,2,\ldots ,N_{g}.$$

The parameter *ψ* determines the shape of the distribution of SNP effect sizes, with larger values of *ψ* concentrating the total effect over fewer SNPs. At *ψ* = 0, all SNPs have the same effect size. Dividing by *N*_*g*_ ensures that the SNP attractor strength distribution defined by Λ_*i*_ is invariant with respect to the number of SNPs. We chose the exponential function for the distribution of SNP effect sizes because it instantiates key characteristics of the current thinking about polygenicity and SNP effects (e.g., Park et al., 2011; Boyle et al., 2017; Holland et al., 2020). In particular, the exponential distribution (1) allows for every SNP to have some effect, (2) can concentrate most of the SNP effects among a small number of SNPs, and (3) lets the amount of SNP effect concentration be a tunable parameter with *ψ* = 0 reducing to uniform SNP effects and *ψ* = ∞ reducing to a single SNP effect.

Each SNP *i* in addition to additive effects, has probability *v* of including a G×E effect Γ_h,i_(*t*) that changes over time *t* depending on the SNP’s proximity to dynamic environment *h*.

(2) $${\text{Γ}}_{\text{h},\text{i}}(t)=\frac{s_{i,h}}{\left|g_{i}-d_{h}(t)\right|{}^{\xi }},s_{i,h}\in \{0,1\},\;P\left(s_{i,h}=1\right)=v,$$

The numerator of Γ_h,i_(*t*) is 1 for SNPs with G×E effects. The denominator of Γ_h,i_(*t*) is a function of the “distance” between the SNP and the dynamic environment. The exponent *ξ* defines the G×E activation curve, or how quickly G×E effects increase as dynamic environment *h* approaches an interacting SNP *i*. Setting *ξ* = 2 yields an inverse square law of attraction similar to gravitational attraction proportional to the inverse of the squared distance between the SNP and the dynamic environment. The notion of proximity or distance used here is abstract and represents the degree to which an environmental factor is in any sense present to the individual, rather than any literal, physical distance. For example, certain individuals may have a heightened genetic liability toward alcohol addiction that does not actively affect their alcoholism or drinking behavior phenotypes until alcohol is initially consumed. The moment at which alcohol is consumed may be regarded in our simulation as a point of close proximity between the dynamic environment representing alcohol and the SNP effect representing the liability.

The G×E that occurs in our model differs from G×E that occurs in some other settings (e.g., Neale & Maes, 2004, Ch. 9). In particular, Equation 2 specifies the moderation (i.e., the suppression or activation) of SNP effects nonlinearly in relation to the distance between the SNP and the dynamic environmental factors. By contrast, G×E often refers to a gene effect that differs as a linear function of the environment (Purcell, 2002). In this latter case, the G×E is nonlinear, but reduces to a a linear gene effect that changes as a linear function of the environment. In our model, the G×E is nonlinear and cannot be reduced to a linear gene effect that changes as a linear function of the environment. Rather, the G×E nonlinearly activates and suppresses gene effects proportional to an inverse power *ξ* of the distance between the SNP and the dynamic environmental factors.

The phenotype-gene-environment system is modeled as as a system of second-order, nonlinear differential equations. The trajectories of the expressed phenotype *p*(*t*) and the dynamic environmental factors *d*_*h*_(*t*) are solutions of these differential equations. In Equation 3, there are five additive terms that give the acceleration $\ddot{p}(t)$ of the phenotype. These terms instantiate the rules about the phenotype-gene-environment system proposed by Turkheimer & Gottesman (1996) and Turkheimer (2004). The first term gives the additive genetic effects and is proportional to the distance between the genes and the phenotype, *g_i_* − *p*(*t*). This term instantiates the first rule that the phenotype is attracted to the genes. The second term gives the G×E and has time-varying effects modified by the gene-environment distance related to *Γ_h,i_*(*t*). This term instantiates the fourth rule that the relative strength of each gene depends on the location of the environment. The third and fourth terms are proportional to the distance between the phenotype and the dynamic and static environmental factors, respectively. This term instantiates the second rule that the phenotype is attracted to the environment. Finally, the fifth term gives viscous friction for the phenotype that resists all movement. This term does not instantiate any rule listed previously, and its role will be discussed later. The third rule is not instantiated in Equation 3, but rather in Equation 7 which states that the environment is attracted to the phenotype.

(3) $$\ddot{p}(t)=\overset{N_{g}}{\underset{i}{\Sigma }}\left[\frac{\alpha }{A}{\text{Λ}}_{i}\left[g_{i}-p(t)\right]+\frac{\gamma }{B(t)}\overset{N_{d}}{\underset{h}{\Sigma }}\Gamma_{h,i}(t)\left[l_{h,i}-p(t)\right]\right]\;+\frac{\delta }{N_{d}}\overset{N_{d}}{\underset{h}{\Sigma }}\left[d_{h}(t)-p(t)\right]+\frac{\epsilon }{N_{e}}\overset{N_{e}}{\underset{j}{\Sigma }}\left[e_{j}-p(t)\right]+\zeta_{p}\dot{p}(t),$$

(4) $$l_{h,i}∼U\left(\left\{0,1,2\right\}\right).$$

Thus, movement of the expressed phenotype *p*(*t*) around its space of possible values is modeled in terms of acceleration $\ddot{p}(t)$ with respect to its current value *p*(*t*), its velocity $\dot{p}(t)$, the current location of dynamic environmental factors *d*_*h*_(*t*), the static SNPS *g_i_* and the static environmental factors *e*_*j*_. The total additive genetic effect is given by *α*, and the total G×E effects is given by γ. *δ* and *∈* give the effect size of the dynamic and static environmental factors, respectively.

The locus of each SNP effect *g*_*i*_ in the phenotypic space is simply the number of minor alleles (i.e., 0, 1, or 2). The linear component of the attraction strength to that locus is then given by ʌ_*i*_, whereas the G×E component is given by Γ_*h,i*_(*t*). The gene-by-environment interaction, with strength given by Γ_*h,t*_, is defined by attraction to a randomly chosen locus in the phenotypic space, *l*_*h,i*_ ( {0,1,2}. Choosing a random point of attraction, rather than the allelic value of the locus activating the G×E effect, ensures that G×E does not simply bifurcate the phenotypic distribution. The normalizing values *A* and *B*(*t*) in Equation 3 are the sums of the additive genetic and G×E effects over all indices of SNPs *i* and dynamic environmental factors *h*:

(5) $$A=\sum_{i}^{N_{g}}{\text{Λ}}_{i}$$

(6) $$B\left(t\right)=\sum_{i}^{N_{g}}\sum_{h}^{N_{d}}\Gamma_{h,i}\left(t\right)$$

Normalizing each set of effect sizes is required to parameterize the model in terms of the total additive genetic effects and G×E on the phenotype, *α* and *γ*, respectively.

Each dynamic environment *d*_*h*_(*t*), in turn, is attracted toward the phenotype with strength *β_h_*:

(7) $${\ddot{d}}_{h}\left(t\right)=\beta_{h}\left[p\left(t\right)-d_{h}\left(t\right)\right]+\zeta_{d}{\dot{d}}_{h}\left(t\right)$$

Equations 3 and 7 together form the system of coupled, nonlinear differential equations that govern the time-evolving dynamics of the phenotype-gene-environment system.

The damping coefficients, *ζ*_*p*_ and *ζ_d_*, in both the phenotypic and environment equations above are included to induce long-term convergence of the phenotype and dynamic environment to fixed values. In physics, damping terms are used to model the dissipation of energy due to friction, and we use this as an analogy for the effects of aging on developmental processes. For example, most of the intraindividual changes in measures of personality occur before age 30 (Costa, et al., 2019). Similarly, it has been reported that older adults aged 70–90 years show significantly lower short-term variability in affect than young adults aged 20–30 years (Röcke, et al., 2009). Neurocognitive functioning has also been found to improve and stabilize into middle adulthood with brain development (Roalf et al., 2014). Our use of damping can also characterize age-related decline in behavioral variation related to cognitive flexibility and learning (Gopnik et al., 2017).

For simplicity, common environment main effects (i.e., *e_j_* values shared across people) were not included in the model. Substantively, this is justified by the infrequency of common environment main effects in most phenotypes, but also allowed us to simplify our examination to just the effects of gene-by-environment interaction. Hence, no main effects of C were simulated.

In the data generation, each person has a series of individual-level components, all of which are perfectly known. The person has a gene sequence of 0s, 1s, and 2s which represent allelic values for each gene location. The person has a time-varying gene activation, environment, and phenotype. Importantly, the gene sequence for each person is constant, but its activation is time varying. In many cases, the phenotype stabilizes to a single steady state value. Therefore, the last time point of the phenotype is considered “the phenotype” unless otherwise stated. Phenotypes that do not converge to a single value might be chaotic or periodic, and consequently might be far less predictable. These phenotypes contribute additional noise to estimates of the heritability. However, the vast majority of systems in our simulation conditions stabilized. A measure of the stabilization is the reduction of variance between the early time points and the later time points. In our simulation study 1, for example, taking the ratio of the variance from the last 50 time points to the variance of the first 50 time points for each phenotypic time series yielded a median value of 0.04 and a 75th percentile of 0.08. This means that the variance at the end of each time series was typically less than 10% of the initial variance.

**Figure 1.**
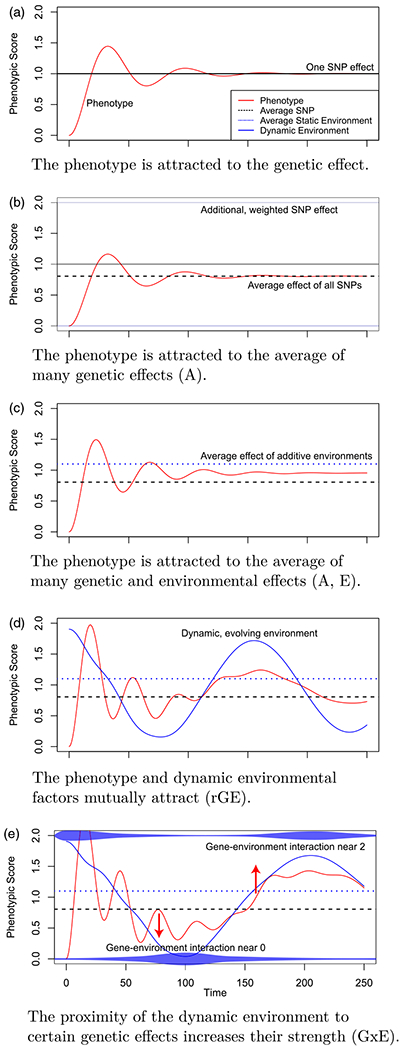
Components of the dynamical gene-by-environment simulation, adding complexity from top to bottom.

Step-wise illustrations of the simulation are shown in Figure 1, starting from the simplest single-gene model with no G×E or other environmental aspects (Figure 1a), and culminating in a system with elements of rGE and G×E (Figure 1e). The path of the phenotype oscillates as it overshoots and corrects around the asymptotic value driven by the SNP effect. The tension between multiple SNP effects results in an asymptotic value at their weighted mean. Static, additive environmental effects behave similarly. In Figure 1d, the presence of a dynamic environment pulls the phenotype in different directions over time. In Figure 1e, the dynamic environment interacts with the genetic effects at 0 and 2, causing a larger downward then upward pull toward those SNP effects when the environment approaches them.

In general, the model developed here can replicate the findings of Turkheimer and Gottesman (1996) and Turkheimer (2004). However, rather than a simple replication of previous findings, we seek to extend the previous findings to modern molecular genetics methods and designs. To assist researchers in their own explorations of these concepts, we provide an online graphical interface for the model at klmckee.shinyapps.io/gxesim (McKee & Hunter, 2021). The graphical app allows setting of all the parameters given in Table 1 and downloading of simulated data.^2^

### Nonlinear phenomena

**Figure 2.**
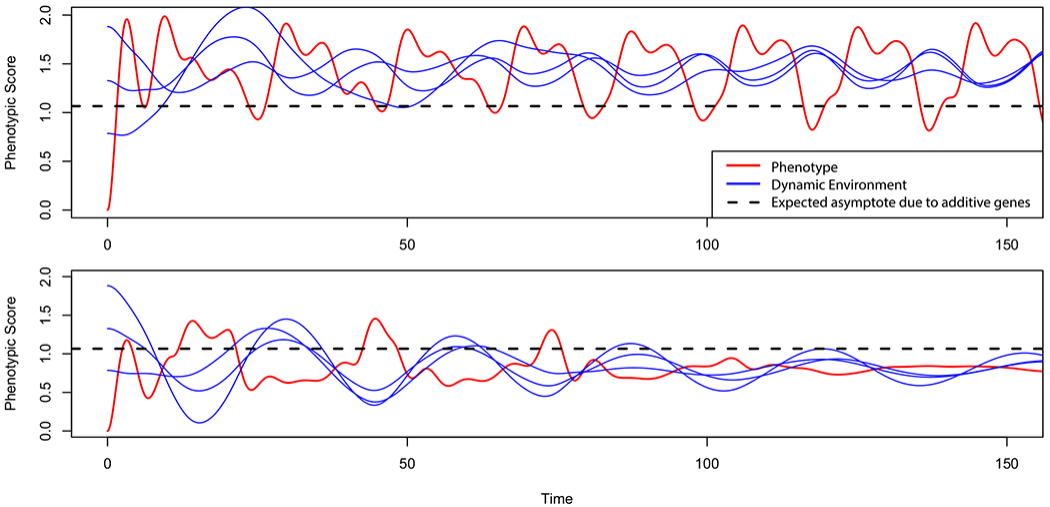
Example simulated monozygotic twin pair phenotypes (red) with identical genomes, identical initial conditions, but different dynamic environmental factors (blue). The Twin 2 phenotype (bottom) converges to the additive genetic expectation. The Twin 1 phenotype (top) does not converge to the additive genetic expectation, but cycles instead. Gene-by-environment interactions result in (1) divergent phenotypic outcomes, (2) different developmental dynamics, with Twin 1 (top) showing periodic change, and (3) deviation from the expected phenotypic outcome under an additive genetic model (dashed line).

When nonlinear mechanisms such as gene-by-environment interaction are introduced into our simulations, many interesting phenomena emerge that resemble observed patterns of development. To demonstrate such variation exclusively as a function of G×E, we simulated monozygotic twin pairs in which all factors were held equivalent except for randomly generated attraction strengths of the dynamic environmental factors and the randomly chosen subset of genes with which they interact. Three dynamic environmental factors were generated per simulation to represent the real complexity and potentially chaotic results of environmental factors. Figure 2 depicts differences in developmental trajectories within a single twin pair and outcomes possible entirely as a result of varied, nonshared gene-by-environment interactions.

**Figure 3.**
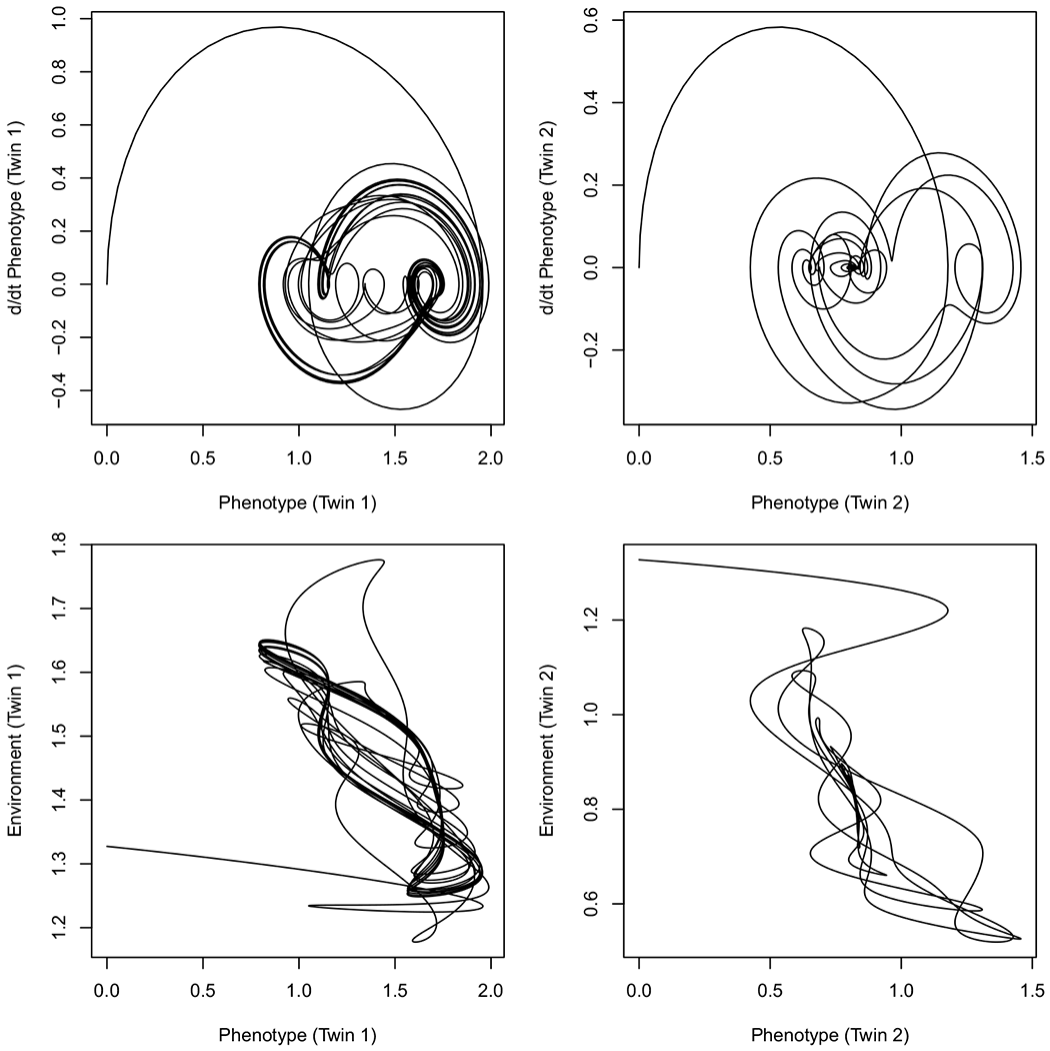
Phase portraits of simulated monozygotic twin pair from Figure 2. Twin 1 converges to a limit cycle, as shown by the trajectory re-tracing nearly the same, looping path repeatedly. Twin 2 converges to a fixed point, as shown by the trajectory settling to a single value.

Although both twins show their greatest unpredictability early on, the most obvious difference is that variation in Twin 2’s phenotype dropped as it approached its asymptote, whereas Twin 1’s phenotype does not converge at all. Rather, it gradually locks into a perpetual limit cycle. Phase portraits of the same trajectories (i.e., bivariate plots of the phenotypes against their instantaneous velocity) are given in Figure 3. Twin 2’s phenotype spirals into a single point value, whereas Twin 1’s phenotype eventually follows a fixed path. The same relationship can be seen between the phenotype and environment, where all three environmental factors gradually change their frequency and phase until they are locked into a mutual orbit with the phenotype.

Figure 3 shows how the synchronization of environmental and phenotypic variation is a consequence of the gradual selection of environmental factors to best accommodate the phenotype over the course of a lifespan. Conversely, the phenotype continues to change to accommodate the environmental influence as well. The resulting selection of environments according to genotype is known as gene–environment correlation, or rGE. Jaffee & Price (2007) comprehensively reviewed rGE and provided numerous examples related to mental illness. For instance, rGE is associated with substance use in which individuals possess both a genetic liability to continue using a drug, and consequently choose peer groups that reinforce that liability (see also, Harden, et al., 2008; Hicks et al., 2013).

## Simulation studies

To gain insights from the proposed model, we conduct four simulation studies. Many simulation studies in behavior genetics begin with assuming the true, data-generating model is closely related to the model used to analyze data, usually variance component models and regression models. We diverge from this path. We begin with the dynamical system model discussed in the previous sections rather than a statistical model. We intend the dynamical system model to have some basic plausibility and correspondence to an abstract description of the true mechanisms which generate gene-environment-phenotype data. We then explore the output from the dynamical system model for its implications on the conventional data-analytic statistical models. The approach is similar to that recently outlined by Borsboom et al. (2021), but has existed elsewhere for decades (see e.g., Atkinson, 1969; Epstein, 1999; Trucano et al., 2006). In studies 1 and 2, we generate data under varying amounts of G×E and apply conventional methods for genome-wide association studies and polygenic risk scores, respectively. In studies 3 and 4, we examine how G×E and relatedness, respectively, impact estimates of heritability from genome-wide complex trait analysis.

### Study 1: Gene finding

Our first study explores under what conditions our model allows individual gene effects to be discovered. In overview, we generate data from our model under three conditions and analyze these data using standard methodology developed for genome-wide association studies (GWAS). The goal is to correctly recover the linear genetic effects in the presence of varying amounts of G×E.

We generate data on 10,000 individuals each with 1,000 genes. All individuals are generated independently, with identity-by-state (IBS) relatedness occurring by chance. The true, linear gene effect sizes follow an exponential distribution as in Equation 1 with *ψ* as in Table 1: a small number of genes have relatively strong effects and the vast majority of genes have small but nonzero effect; all genes are at least somewhat associated with the phenotype. We vary the strength of the G×E effect size *γ* from Equation 3 across conditions. In condition (a), the G×E effect strength is equal to the additive genetic effect strength: *γ* = *α* = 0.05. In condition (b), the G×E effect strength is slightly larger than the additive genetic effect strength: *γ* = 0.20, *α* = 0.05. In condition (c), the G×E effect strength is much larger than the linear effect strength: *γ* = 0.35, *α* = 0.05. These three conditions create models that are (a) almost linear, (b) moderately nonlinear, or (c) highly nonlinear.

**Figure 4.**
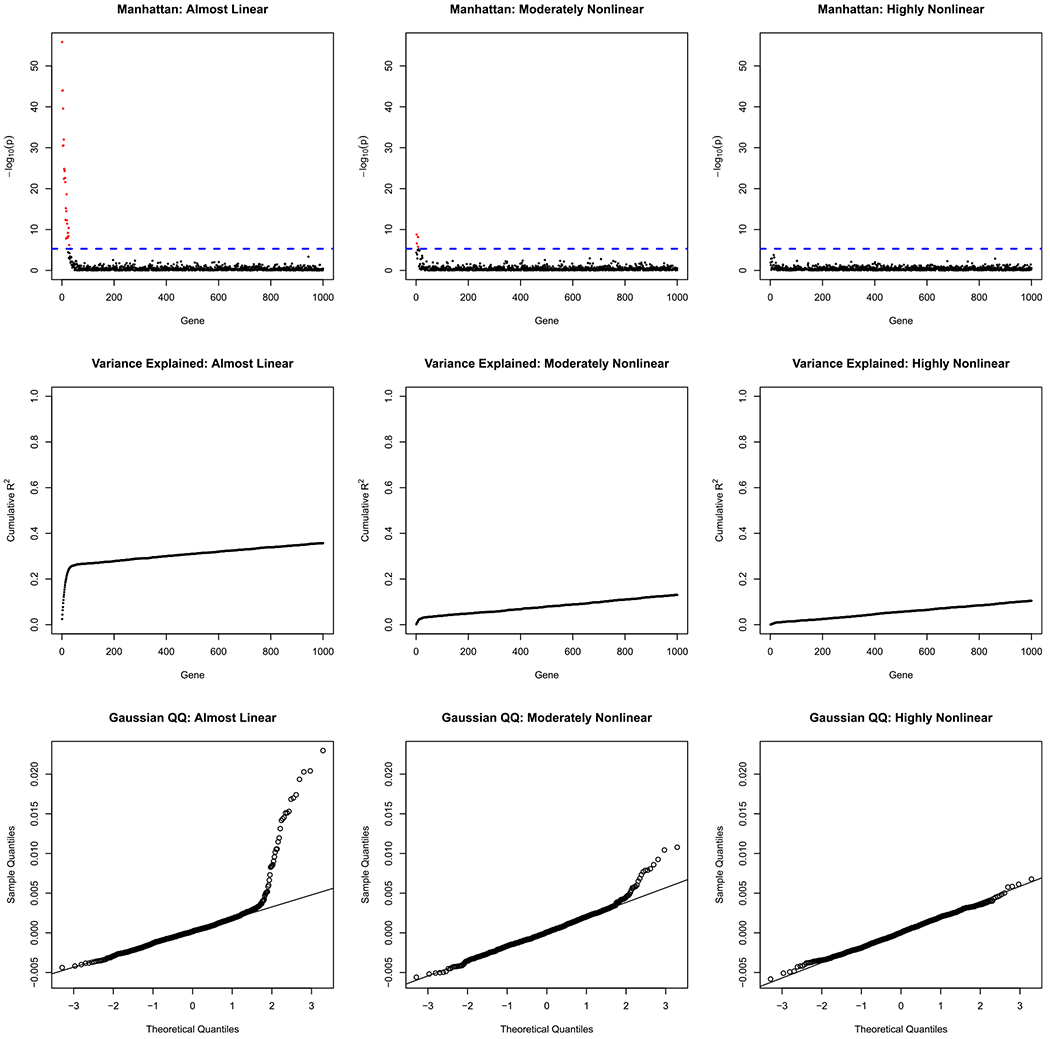
Manhattan, cumulative variance explained by genes, and QQ plots for Study 1. Rows have different kinds of outcome measures. Columns have different conditions.

We analyze the data with standard GWAS methods. In particular, we predict the phenotype at the last time point from the gene at index location *i* for all possible locations using ordinary least squares regression. From the regressions, we obtain the slopes, squared correlations (*R*^2^), and *p*-values for each of the 1,000 genes. Each of these three outcomes is shown for each of the three conditions in Figure 4.

In the first row of Figure 4, the number of leading zeros in the *p*-value (i.e., minus logarithm base 10) is graphed against the gene index to create a pseudo Manhattan plot. We call the first row of 4 a pseudo Manhattan plot because there is no true sense of gene location in our simulated genome. Genes that are statistically significant after a Bonferroni correction are highlighted as being above the dashed line. In the “almost linear” condition, 26 genes have Bonferroni-corrected significant effects, and the genes with the largest true effects are the ones that are observed empirically to have the largest effects. In the “moderately nonlinear” condition, only 4 genes have Bonferroni-corrected significant effects. In the “highly nonlinear” condition, no genes have Bonferroni-corrected significant effects. So, in the almost linear condition, the linear part of the true gene effects can be recovered, but nonlinearity destroys the ability to discover the linear gene effects.^3^ As the strength of the nonlinear effect increases, the ability to find statistically significant effects for genes decreases.

In the second row of Figure 4, the cumulative variance explained by the genes (*R*^2^) is plotted against the gene index. The cumulative *R*^2^ is computed by summing the *R*^2^ from each of the separate simple linear regressions. Therefore, it is theoretically possible for the cumulative variance to exceed the total phenotypic variance due to correlated predictors being entered as predictors in separate regressions with a common outcome variable.^4^ However, even in the almost linear condition, the cumulative *R*^2^ never exceeds 0.40 in these simulated data because the true gene effects are independent. Across all conditions, the first few genes have the largest effects and thus increase the cumulative *R*^2^ at a steep rate, whereas the later genes have smaller effects and thus increase the cumulative *R*^2^ at a shallow rate. As the condition changes from almost linear to highly nonlinear, the cumulative *R*^2^ decreases. The same linear gene effects have different *R*^2^ values depending on the strength of the nonlinearity. Hence, the nonlinearity destroys the ability of the linear gene effects to explain phenotypic variance. Whereas the first row of Figure 4 shows the effect of nonlinearity on statistical significance, the second row of Figure 4 shows the effect of nonlinearity on effect size.

In the third row of Figure 4, the estimated gene slope effects are compared to a Gaussian distribution in a QQ plot. Gene slope effects that are purely Gaussian follow the line added to each panel and suggest the effects are not real gene effects, but merely capitalize on chance relationships due to sampling variability. Again, in the almost linear case, there is clear evidence of real, linear gene effects as indicated by the strong deviation from a Gaussian distribution. However, as the nonlinearity increases, the linear gene effects become indistinguishable from Gaussian noise. Therefore, nonlinearity destroys the ability to separate true, linear gene effects from random noise gene effects.

Taken together, study 1 finds that when the true situation is primarily governed by linear gene effects, then the typical gene-finding methods in GWAS do indeed find the correct, linear gene effects. However, as nonlinearity increases, the true, linear gene effects that are present are no longer discoverable. These findings hold regardless of the metric used for gene finding: Manhattan plot, cumulative variance explained by genes, or QQ plot of the SNP effects. Given the results of study 1 for GWAS, we expect similar findings for risk prediction using polygenic risk scores and for heritability estimation using genome-wide complex trait analysis. However, to eliminate the possibility of some unexpected results, we still conduct their analyses.

### Study 2: Risk prediction

In study 2 we use the same data generated in study 1, but for a new purpose. Instead of attempting to find the genes that have the largest influences on the phenotype, we are now attempting to explain as much variability in the phenotype as possible while using only the SNPs. The method creates a linear regression model with the phenotype as the outcome and some – but usually not all – of the SNPs as predictors. The regression model outputs a “risk” score for the phenotype (i.e., the predicted value of the phenotype for continuous phenotypes, or the forecasted probability of a binary phenotype) based on many, linear gene effects. Thus, the predicted phenotype is called a polygenic risk score (PRS).

The PRS differs from the GWAS in several important ways. First, GWAS is often a preliminary step to PRS regression that determines which and how many SNPs are used to create the PRS. Second, multiple SNP effects are used simultaneously to predict the phenotype in PRS regression, whereas GWAS exclusively considers one SNP at a time. Third, the evaluation of a PRS often uses a hold-out sample: that is, the PRS model is trained on one part of a data set, but is then evaluated on a separate part of the data set that was not used to fit the model. This cross-validation is critical to PRS evaluation. In study 2, we evaluate the utility of PRS under the same nonlinearity conditions as study 1 using the same data, but now with the addition of multiple regression and cross-validation.

**Figure 5.**
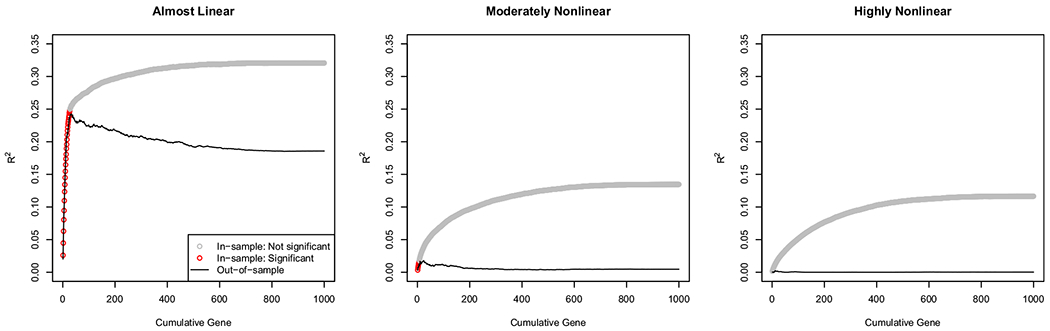
Cross-validated *R*^2^ for polygenic risk scores created by adding SNPs in order of statistical significance. A point is highlighted in red if the corresponding SNP effect is statistically significant at the Bonferroni corrected level. Note that *R*^2^ is theoretically between 0 and 1, but the vertical axis range is restricted.

Figure 5 shows the cross-validated *R*^2^ for a PRS created by adding SNP predictors cumulatively in order from most statistically significant to least statistically significant. Thus, the first PRS uses the single SNP with the smallest *p*-value; the second PRS uses the two SNPs with smallest *p*-values; and so on until the final PRS uses all available SNPs. The gray points indicate the *R*^2^ for the in-sample data that trained the model, thus being vulnerable to capitalization on chance. The red points are Bonferroni-corrected statistically significant. The black line indicates the out-of-sample data, thus being a measure of predictive quality that is less susceptible to capitalization on chance.

In the almost linear condition, there is clear predictive utility to the PRS for the first several SNPs. However, after the first 50 SNPs, the out-of-sample *R*^2^ decreases. The peak *R*^2^ in the almost linear condition is slightly more than 0.20 which matches the findings observed in many studies. In the moderately nonlinear and highly nonlinear conditions, the in-sample *R*^2^ follows a similar pattern to the almost linear condition: rapid initial increase, followed by an asymptote. However, the out-of-sample *R*^2^ never shows any appreciable predictive utility. Note that the shared vertical scale across conditions obscures some of the nuances for the moderately nonlinear and highly nonlinear conditions.

The findings for study 2 replicate those of study 1. In the almost linear condition, a useful PRS is possible to construct. The predictive utility is limited by the small amount of nonlinearity that is present, but a high-quality PRS can explain around 20 percent of the variance in the phenotype using only a linear combination of the SNPs. However, as the nonlinear effect strength *γ* increases, all predictive quality of the PRS is eliminated.

### Study 3: Heritability and nonlinearity

In study 3, we simulated data to determine the impact of G×E on the ability to recover the heritability of a phenotype from a sample of people with measured genomes. In assessing heritability, we want to know how much variability can be explained by all the gene effects together. We report narrow sense heritability as a value between 0 and 1, the proportion of phenotypic variance accounted for by the linear, additive effect of genes. Methods for estimating heritability from samples of known relatives have been well-known for one hundred years (e.g., Wright, 1920), but these have only recently been updated to use nominally unrelated people with measured genomes. Yang, et al., Visscher (2011) developed the primary method for estimating heritability from measured genomic data, genome-wide complex trait analysis (GCTA). GCTA uses the observed pairwise similarity of all the individuals’ genomes in a sample to explain respective pairwise similarities among phenotypic scores. Mathematically, GCTA is a multilevel model (e.g., Laird & Ware, 1982; Pinheiro & Bates, 2000; Snijders & Bosker, 2011) with one large “family” acting as a single level 2 unit and all the distantly-related people acting as level 1 units nested within the “family” (cf. Laird & Ware, 1982, Equation 2.1 with Yang et al., 2011, Equation 1). Furthermore, whereas the subjects of family studies are related according to identity-by-descent (IBD), the correlations used in GCTA represent identity-by-state (IBS) relatedness, or the chance coincidence of alleles among unrelated subjects. In a standard GCTA, heritabiity is the proportion of the total phenotypic variance attributed to additive genetic random effects. The correlation structure of the genetic random effects is set to the correlations of genomes computed *a priori* between pairs of unrelated individuals, which are usually between plus and minus 0.025. This correlation structure gives the IBS genetic relatedness between people.

GCTA differs from GWAS and PRS in several important ways. Unlike GWAS and PRS, GCTA does not seek particular genes that influence the phenotype. Rather, GCTA estimates the proportion of variability “explained” by genes without any effort to determine the loci or magnitude of particular effects. Moreover, GCTA does not predict particular phenotypes or evaluate the “risk” of developing a phenotypic outcome. Instead, GCTA addresses the question of the total, additive genetic contribution to phenotypic variation.

In the simulation for study 3, the goal was to vary the strength of G×E and observe its impact on estimates of heritability obtained by GCTA. We simulated 2000 independent and nominally unrelated genomes and corresponding phenotypic trajectories for each condition. The resulting SNP correlations had similar properties to those commonly found with real SNP correlations (e.g., mean of zero, approximately Gaussian distribution, and most SNP correlations between −0.025 and 0.025). Environmental dynamics (*δ*, *d*_0_) were randomly generated and unique to each individual. The G×E effect size (*γ*) was gradually increased from 0, in which case genetic effects were entirely additive and the GCTA model was correctly specified, to 0.2, simulating strong, unmodeled G×E. The total additive genetic attractor strength (*α* was fixed at 0.05, and the additive, static environmental effect *∈* was set to 0.01 for one random static environmental attractor. In this simulation, static genetic and environmental effects were present and expected to be accurately represented by GCTA estimates under the first simulated condition in which G×E effects *γ* = 0. Estimates from subsequent conditions with non-zero G×E thus used estimates from the first condition as the point of comparison.

**Figure 6.**
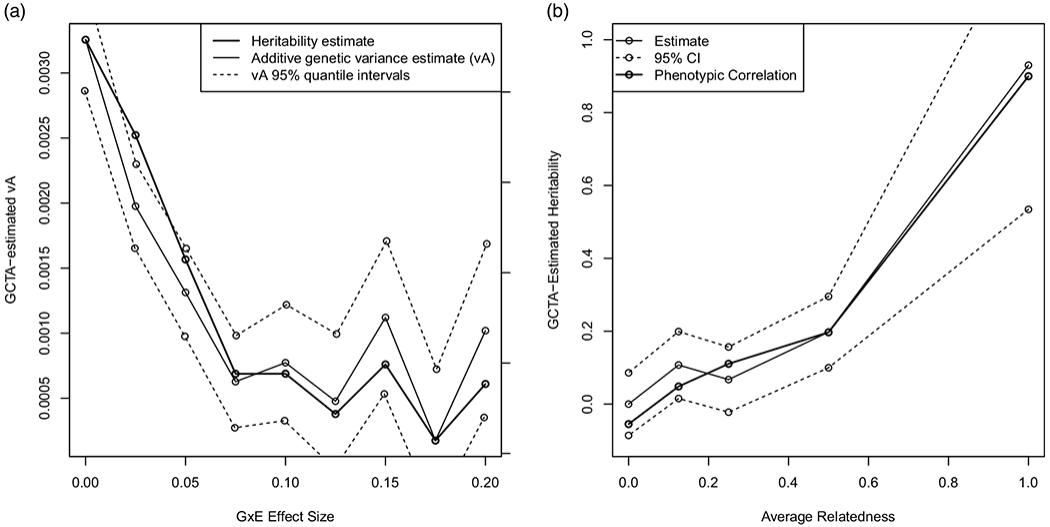
GCTA Simulations with gene-by-environment interaction. Ninety-five percent confidence intervals are shown as dotted lines.

Figure 6a shows the estimates of heritability for each simulated value of *γ*. As the data incorporate larger Gx×E effects, the estimated heritability decreases even though the total strength of the static, additive genetic effects is unchanged. Within each condition, the effect of genes varies as a function of the environment; however, across conditions, the additive genetic effects are constant. In such a situation, it would be desirable for GCTA to recover the same additive genetic effect across conditions. To the contrary, the estimated heritability in the reference condition (i.e., with no G×E) is about 80 percent, but as G×E increases, the estimated heritability approaches its lower limit of zero. The change in heritability cannot be explained by the addition of environmental variance alone. That is, the proportion of the additive genetic variance cannot be explained by simply increasing the total variance (e.g., by increasing the environmental variance). As Figure 6a shows, the raw estimates of additive genetic variance on the phenotypic scale reduce with the G×E effect, not merely as a proportion of total variance. We interpret this trend as the presence of G×E in the data obscuring the static, additive genetic effects in the estimated heritability. The situation is akin to fitting a regression model with an interaction term, and finding that the size of the main effect estimate changed when the size of the interaction coefficient changed: theoretically, the main effect and interaction coefficients should not influence each other. Thus, Figure 6a does not show a decrease in the true genetic effect, but rather a decrease in the estimated contribution of genetics due to G×E. Sufficient nonlinearity precludes the ability to recover heritability, even when we know it is present.

### Study 4: Heritability and relatedness

In our fourth and final study, we simulated data to explore how the degree of genetic relatedness among pairs of participants in a sample impacts the estimates of heritability when an interaction between genes and the shared environment is present. A well-established finding is that the heritability estimates from twin and family designs are often larger than those found using GCTA with nominally unrelated people. The deficit in the estimated heritability among unrelated people is called missing heritability, and holds regardless of the phenotype. We use the same GCTA method in study 4 as study 3, but instead of varying the G×E effect strength, we vary the degree of relatedness among pairs of simulated people. The GCTA then uses both within-family and between-family genetic correlations. The within-family correlation, or relatedness, varies by condition across typical familial values (i.e., 0.125 for cousins, and 0.5 for full siblings). The between-family relatedness is for unrelated individuals and is always near zero (i.e., usually plus or minus 0.025).

Twin and family designs of closely related people (usually parent-child pairs, sibling pairs, or twin pairs) can be seen as a special case of the modern measured genome designs of nominally unrelated people (Hunter, 2021; Hunter, et al., 2021). The differences are (1) use of *a priori* relatedness given by the zygosity of the relatives, such as a genetic correlation of 1.0 in monozygotic twins and 0.5 in dizygotic twins, versus empirical genetic correlations in unrelated individuals, (2) the size of the family, which is usually two in twin and family designs, but thousands in the modern measured genome designs, and (3) how relatedness is measured, which is usually average amount of segregating genes shared in twin and family designs (identity by descent, IBD), but the measured correlation between SNPs in modern molecular designs (identity by state, IBS). Hence, missing heritability could arise as a function of pair relatedness in the sample or as a function of differences between IBD and IBS relatedness (see Young, 2019, for a detailed discussion of both mechanisms). In study 4, we investigate the role pair relatedness could play in missing heritability.

We propose that one source of missing heritability specifically occurs because shared genes interact with shared environmental factors (i.e, A–C interaction). If an A–C interaction was present but was omitted from the fitted model, the effect of the genes could be systematically incorrect (i.e., the variance of A could be biased; Eaves, et al., 1977; Eaves, et al., 1978; Keller & Coventry, 2005). The size of this interaction is captured by the G×E effect strength (γ) in our model. By simulating samples for GCTA in which phenotypic scores are subject to gene-by-shared-environment (G×C) interaction and varying degrees of relatedness between pairs of participants, we can examine the emergence of missing heritability on a continuum relating the typical results of GCTA to those of twin models.

In the study 4 simulation, the sample consisted of 1000 simulated pairs of relatives, with related individuals sharing both the initial values and dynamics of their environmental influences, representing familial environment. Related genomes were generated by first producing two random, independent genomes, then randomly replacing the specified proportion (i.e., the relatedness coefficient) of alleles in one person’s genome with the relative’s values at the same loci. No environmental attributes (dynamics or start values) or genes were shared between pairs. The GCTA estimates of heritability used empirical, IBS estimates of both the correlations within and between pairs, rather than including the relatedness *a priori* via IBD as in a twin or family model. Thus, the nominal relatedness within families (e.g., 0.125 for cousins) slightly differed across families (i.e., varied due to sampling variability by amounts typically found with IBS estimates of close relatives).

The gene-by-environment effect size was fixed at the highest value from the study 3 simulation, *γ* = 0.2. All other parameters remained the same as those in study 3, with the exception that dynamic environmental factors were constrained to have the same dynamic properties and gene pairings among related twins. The degree of relatedness among the pairs varied across conditions: 0 (unrelated), 0.125 (first cousins), 0.25 (half siblings), 0.5 (DZ twins or full siblings), and 1.0 (MZ twins). In each condition, the sample consisted entirely of people with only one type of relatedness. Thus, the first condition has only first cousins; the second condition has only half siblings; and so on. The goal was to see the effect of the degree of relatedness on estimated heritability in the presence of gene-by-environment interaction when related individuals also shared the environmental component of the interaction. The model contained no specific sources of linear shared environmental effects (C), as is typically the case for GCTA studies of unrelated individuals.

Figure 6b shows GCTA-estimated heritability across relatedness conditions. In samples of more closely related pairs (e.g. MZ twins), GCTA-estimated heritability is higher than in samples of more distantly related pairs (e.g., first cousins). Even though the true genetic and environmental effect strengths were the same across conditions, GCTA-estimated heritability was about 0.93 among the MZ twin pairs, but less than half of that (0.19) for sibling pairs (relatedness of 0.5). The phenotypic correlation is also shown, with little difference from the estimated heritability as only a small amount of unique environmental variance (E) was simulated. Thus, estimates of heritability differ purely as a function of degree of relatedness. Therefore, the missing heritability of GCTA methods may be due purely to the samples that GCTA typically uses which have lower degrees of relatedness. Our model suggests that missing heritability could be a consequence of the combination of (1) lower degrees of relatedness in GCTA analyses than twin analyses and (2) the existence of nonlinear gene-by-environment interaction effects. That is, our model replicates an observed pattern of missing heritability similar to what we find in real data, and the causal factors in our simulation are the degree of relatedness and the nonlinear interaction effects. Whether or not these causes operate in real data remains an open question, but we suggest these mechanisms for further investigation.

## Discussion

Twenty years ago, the senior author demonstrated that a simple dynamic simulation of genes and environment could reproduce some of the more perplexing findings of twin studies. Here, we have shown that a more sophisticated version of the same basic principles can reproduce many of the findings that have occurred in complex genetics since that time, especially as regards analysis of measured DNA in place of indirect inferences from twins. We have proposed an abstract dynamical systems model of phenotypic development involving gene-by-environment interaction and gene-by-environment correlation. Model simulations were used to produce phenotypic distributions under various conditions, which could then be used to test theoretical and methodological questions about nonlinear development.

In the first and second set of simulations, we considered recovery of SNP effects in GWAS and the utility of corresponding polygenic risk scores in the presence of gene-by-environment interaction. In a linear model with unrelated individuals and thus no shared environmental effects, GxE contributes uniquely to each individual due to the dependence on environmental factors. Adding G×E to the simulation not only introduced effects that could not be subsequently recovered, but also demonstrated masking of otherwise recoverable linear SNP effects.

In the third and fourth set of simulations, we considered recovery of total heritability estimated with GCTA in the presence of gene-by-environment interaction and varying degrees of relatedness in the sample. G×E decreased GCTA-based estimates of heritability to near zero even when the simulated genetic effects accounted for 80% of the total variance. However, some portion of the genetic effect can still be recovered in GCTA when the sample includes many pairs or groups of related individuals that share not only genes but environmental factors. This result gives one possible explanation for why twin studies produce larger estimates of heritability overall than GCTA or polygenic risk scores from GWAS.

### Implications

#### Classical twin models

In terms of latent variance components, notated A for additive genetic, C for common or shared environmental, and E for unique environmental variance, an interaction of genes with shared environment would be an A–C interaction, whereas the more common concept of individual-specific G×E is better described as an A–E interaction. The presence of a true, underlying A–C interaction inflates estimates of A in the classical twin model, but not in models where participants are unrelated, because it depends on the existence of shared environmental factors. However, A–C interaction does not necessitate a main effect from C. An A–C interaction with no main effects may be understood as a shared environmental factor of which the effect is conditional entirely upon particular alleles. In our simulations, no static C-component was simulated, but A–C interaction took the form of a dynamic environmental attractor shared between twins. The effects of particular genes were enhanced by proximity to the dynamic environment, and MZ twins were more likely to share genes that interacted with the dynamic environment. In this way, unmodeled A–C interaction with no main C effect is one possible cause of the disparity in heritability estimates between twin studies, GCTA, and via PRS.

The A–C interaction term can, in principle, be included in a twin and family modeling framework and is statistically identified in several designs, including the commonplace twins reared together and apart design. Hunter et al., (2021) showed both a general method for determining model identification in variance component models and specifically discussed the A–C interaction term in the twins reared apart and together design. The variation in genetic relatedness across zygosity that is separate from the variation in environmental relatedness across twins reared together or apart identifies the A–C interaction. From an A–C interaction in a heritable trait, we expect the phenotypic correlation between members of a twin pair to differ by zygosity, shared rearing environment, and the combination of zygosity and rearing environment. The model can be specified such that the common environmental relatedness coefficient is fixed to zero for all twin pairs reared apart. The A-C interaction term is then given as an additional variance component acting to reduce the predicted phenotypic correlation between MZ twins reared apart and – to a lesser extent – reduce the predicted phenotypic correlation between DZ twins reared apart (see Hunter et al., 2021, p. 430, Equation 21).

A variance-components representation of gene-by-environment interactions dispenses with the chaotic details of exactly how the phenotype is nonlinearly governed by combined genes and environment and refers only the expected distribution of the phenotype taken at some developmental terminus or as a time-invariant abstraction. Other, more specific nonlinear components could be considered possible: for example, an interaction between A and logit^−1^(C), in which case the additive genetic contribution is scaled between 0 and 100% by the common environmental factor, or A^2^C, in which case the effects of the common environmental factor are largest when one falls close to either tail of the additive genetic liability distribution. For most purposes the A–C product adequately represents the property that additional effects on the phenotype arise only as a joint function of both factors.

#### Genes and environmental factors: inherently infinitesimal?

Boyle et al., (2017) observed that Fisher’s infinitesimal model matches observations more than many researchers expected, and hypothesized that large regulatory gene networks could explain why the infinitesimal model so closely corresponds to the observed data. To elaborate, some researchers expected to find a small number of genes (e.g., less than 15) with biologically plausible causal pathways to a phenotype. Moreover, these researchers thought that this small number of genes would explain most of the genetic variability in the phenotype. However, we often find a large number of genes related to a given phenotype, many of which have no biologically plausible connection. Moreover, each of the genes that do have a biologically plausible connection explain only a small portion of phenotypic variability. Our nonlinear gene-environment-phenotype model is a complementary explanation for the same phenomena. By introducing a dynamic gene-by-environment interaction in individual development, a nonlinear gene-environment-phenotype dynamical system can account for some of the same difficulties in uncovering causal alleles as the omnigenics model of Boyle et al., (2017). These two sources of complexity – regulatory networks at a cellular level and dynamic interactions at the level of the organism – are not mutually exclusive, and both are probably at work in the development of complex phenotypes.

The consequence of these considerations is that the original hope of DNA-based complex genetics, that of finding individual genes or SNPs with statistical main effects of sufficient magnitude to form the basis of biological processes leading from the genome to high-level phenotypes, will under a reasonable set of parameters be mostly impossible. It is important to note that the simulations we have performed, while complex in comparison to simple linear models, almost certainly underestimate the complexity of actual human development by many orders of magnitude. If one imagines the kinds of “regulatory networks” that might underlie a trait like human personality, and multiply them by individual-level G×E interactions in the course of behavioral development, one can quickly reach a level of complexity that will not be rescued by ever-larger sample sizes or more sophisticated sequencing.

#### Missing heritability: a call to re-think linearity

In all of our simulation studies, genes had a strong causal influence on the phenotype. However, modern molecular behavioral genetics techniques only found the gene effects when the dynamics were mostly linear (i.e., in the absence of gene-by-environment interaction). We could not find the gene effects using GWAS, PRS, or GCTA even though we knew the effects were present. In classical twin and family designs it is well-known that some nonlinear genetic effects (e.g., dominance) attenuate linear genetic effect sizes (Neale & Maes, 2004). These same attenuation effects hold for modern molecular designs, yet the techniques of the modern genomics era typically ignore all nonlinearity or adjust for only the simplest forms of nonlinearity (e.g., dominance) while ignoring others (e.g., higher-order gene interactions and gene-by-environment interactions). Thus, there are certainly causal genetic mechanisms that are not found because they are nonlinear. The same holds true for environmental causal mechanisms. Although the present work has emphasized discovery of genetic causal mechanisms, the original work along this avenue showed that environmental factors could have causal influences that could not be found outside of genetic clones (Turkheimer & Gottesman, 1996; Turkheimer, 2004). Our simulations suggest that similar non-discoverable causes might exist for genetic mechanisms.

Our study 4 produced one possible explanation for the phenomena of missing heritability: estimates of heritability were higher in samples of more closely related people than in samples of less related people. This simulation study suggests that missing heritability can result from unmodeled A–C interaction. Specifically, people who are closely related may also share many of the same environmental influences, and consequently, may both experience the same gene-by-environment interactions. The kind of heritability detected due to A–C interaction in our simulation is not, however, the same additive source of variation expected by a GWAS or GCTA, and may be regarded as a separate kind of heritability altogether.

It may be argued that GCTA and other linear methods do perform their intended task correctly by only estimating the additive, linear contributions of genes, whereas the missing heritability found in twin models reflects violations of their core assumptions. At its root, the missing heritability issue invokes the question of how heritability is best characterized. For certain purposes, it may not be satisfactory or relevant to define heritability such that it conditions on transient, unpredictable, and mutable environmental factors. If heritability is specifically posed in the context of fixed, immutable sources of variation in the population, then additive linear effects, though potentially very small, are still of central interest. Where additive effects are consistently very small, the utility of such a definition becomes less clear. We argue that when the goal of research is to understand the specific relations between genes and phenotypic outcomes, overly general concepts like heritability are far less useful than specific theories about gene-by-environment complexity.

#### Development is a complex system

It is an historical irony that the field of complex trait genetics has seen little input from the science of complexity. Human development is certainly a complex system in the colloquial sense of a system that is complicated. However, Molenaar, et al., (1993) have argued that human development is necessarily a complex system in the technical sense of a system that is composed of many interacting parts where the interactions are critical in determining the total system’s behavior. Such complex systems have several defining features. In particular, complex systems are nonlinear, selforganizing, and are often governed by a small number critical nonlinearities. For example, complex flocking behavior seen in several species of birds and fish can be explained by a small set of nonlinear rules (Cucker & Smale, 2007). Similarly, atmospheric circulation (i.e., Bénard convection) can be modeled by a simple, nonlinear three-dimensional system (Lorenz, 1963). Our studies suggest that a similarly small set of nonlinear rules can account for several common findings of behavior genetics. Our model proposed one set of rules based on theoretical considerations.

Developmental studies of behaviorally complex traits commonly find that the relative influence of genes is greatest later in life (Haworth et al., 2010; Plomin, et al., 1994), due in part to delayed genetic expression, and in part because most environmental influences are transient. After creating a simple, linear genetic developmental model, Eaves, et al., (1986) also found that even small genetic effects “may have cumulative effects on individual phenotypes that far outweigh the substantial but unsystematic effects of the environment.” (p. 153). Our nonlinear model formalizes long-term mechanisms and outcomes of development as a dynamical system in terms of equilibrium and convergence dynamics. Such a nonlinear modeling perspective enriches our understanding of development by framing it in the context of dynamical systems.

### Limitations

#### Biological fidelity of the model

We developed the mathematical model from several basic principles in biology and behavior genetics. However, the model lacks full fidelity to some known aspects of biology. In particular, the model lacks any representation of chromosomes, inheritance, and consequently linkage and linkage disequilibrium (see Bulik-Sullivan et al., 2015, for how linkage disequilibrium itself can be used to estimate heritability). Furthermore, the model has no way of distinguishing protein-encoding genes from the majority of genes which do not encode proteins but rather are thought to regulate gene expression. Finally, the model also lacks epigenetic mechanisms that can represent a type of G×E: gene expression that is enabled or inhibited by DNA methylation or histone modifications. Although the model lacks these aspects of known biology, we believe the fundamental insights stand regardless. Moreover, the biology could be incorporated into the model with some difficulty but without requiring core changes. For instance, epigenetic mechanisms could be posed exactly as we have specified gene-by-environment interactions, as they generally imply activation and suppression of genetic expression over time conditional on changes in the environment.

#### Phenotypic asymptotes and definitions

For our simulations, we defined “the phenotype” as the value of the time-varying phenotype upon convergence after a sufficient amount of time has passed. This results in relatively simple analysis of the outputs in terms of phenotypic variance components, as all periodic or chaotic variability leading up to that point can be disregarded. However, other questions may be considered or framed using different phenotypic definitions. If the phenotype is defined to be a particular value in the trajectory before long-term convergence, then such periodic or chaotic movement of the dynamic environment behaves as a source of unique environmental variance. Other definitions are possible, such as frequency bands from a spectral analysis of the phenotype or descriptive statistics such as the mean and standard deviation of the total distribution of each person’s time series. For example, the parameters of genetic and environmental complexity in our model may be adjusted to reproduce observed episodic fluctuations observed in bipolar disorder, binging behavior associated with eating disorders, alcohol, or substance abuse. In each of these, the phenotypic variable (*p*(*t*)) may be momentary affect, eating behavior, number of drinks or cigarettes, respectively, while the frequency and amplitude of their fluctuations represent clinically meaningful complex traits, subject to the underlying genetic and environmental architecture.

#### Design choices

It is a common limitation of complex simulations such as this that there are many degrees of freedom that allow the production of nearly any desired result. This simulation involved both many parameters and many possible interpretations of the parameters and the resulting data. For this reason, we chose phenotypic definitions and model parameterizations that (1) did not rely on random noise, (2) represented only key, well-known concepts in behavioral genetics, and (3) represented such concepts with minimal unnecessary complexity.

Other representations of the same concepts would be possible with stochastic simulations. Such simulations would have involved stochastic differential equations (SDEs) instead of the ordinary differential equations (ODEs) used in our model. Some benefit of simulating with SDEs include known analytic derivations of the total series variance and prevention of identical outcomes due to identical starting conditions. However, our simulations demonstrate that the key concepts of behavioral genetics can be represented without random noise, although the complex, deterministic variations in time series here are necessarily subsumed in noise components of corresponding cross-sectional variance-components models.

#### Resemblance to empirical findings

Our simulation serves mainly as a proof of concept and was not intended to replicate any specific phenotypic findings. Our dynamic environmental factors, for example, are particularly simple, taking the form of smooth sinusoidal functions. Many real complex traits exhibit much greater environmental and developmental complexity, and much smaller estimated heritabilities. By keeping the model relatively simple, we found that even a small set of nonlinear factors can create dramatic problems for finding causal effects. Using unrealistic genetic effects allowed us to demonstrate a general principle with minimal unnecessary computation that nonetheless can be scaled to more realistic effects.

### Future Directions

The simulation model can be configured to replicate empirical findings in specific phenotypes, including additive genetic and environmental variance components, nonlinearities, and temporal or developmental patterns at various timescales. If some or all of this information has been determined previously, then the simulation may be used to infer other aspects of the phenotypic model that are difficult or impossible to estimate empirically, either due to a lack of data, determination of covariates or endophenotypes, or because models with necessary components like A–C interaction are difficult or impossible to statistically identify (i.e., solve mathematically to obtain optimal parameter estimates). We have produced an online graphical interface for the model and invite phenotypic domain experts to explore configurations of the model that best represent known phenotypic etiology (McKee & Hunter, 2021).

As this modeling approach is primarily theoretical and differs from conventional data-driven fitting and estimation procedures, we did not design the model for parameter identification. Hence, fitting the simulated trajectories to data would be difficult. If appropriate, momentary phenotypic data were available in abundance over the full developmental timeline, then some parameters of the model could be estimated approximately using continuous-time state-space model techniques. Two such parameters include the attraction between the phenotype and environment (*ϵ*, *δ*) and the damping parameters of each (*ζ_p_*, *ζ_d_*), as these are represented in models of continuous-time vector autoregression. Hunter (2018) and McKee (2021) have published guides to state-space modeling in a variety of software, including OpenMx (Boker et al., 2021; Neale et al., 2016) and Stan (Carpenter et al., 2017). At present, however, it is infeasible to collect phenotypic and environmental scores at sufficient resolution over the lifespan to fully fit the given equations, which were intended as abstract guides to developmental theory.

## Conclusions

Genetic studies of complex traits often show disparities in estimated heritability depending on the method used, whether by modeling twins, GWAS and associated PRS, or by GCTA. We developed a simulation of individual genomes and dynamic environmental conditions to consider how linear effects, gene-by-environment interactions, and gene-by-environment correlations may work together to govern the long-term development of complex traits. Our simulation studies demonstrate ways complexity obscures linear, narrow sense heritability in conventional modeling strategies and produces a more nuanced, nonlinear understanding of genetic effects. The genetic effects estimated by GWAS and GCTA in unrelated individuals were inadequate to characterize the true, data-generating gene-by-environment model.

Inclusion of related individuals in GCTA resulted in high heritability estimates when highly related individuals shared a common dynamic environment and the genes with which it interacts. The resulting estimated heritability represents genetic sources of phenotypic covariance that are distinct from, and can actually mask, additive genetic variance. Researchers are thus likely to underestimate the roles of both genetic and common environmental factors in phenotypic variation when only modeling unrelated individuals from independent environments.

Future studies may use the simulation model to test hypotheses about phenotypic complexity either in a general way or by replicating well-established observations of specific phenotypes.

## Figures and Tables

**Table 1.** Model variables and user-input simulation parameters

Parameter	Function	Default value
*π*	Minor allele frequency (MAF)	0.5
*ψ*	Uniformity of SNP effect sizes	50
*α*	Total additive genetic effect (produces A)	0.05
*ε*	Attraction of phenotype to static, additive environmental factors	0.05
*δ*	Attraction of phenotype to dynamic environmental factors	0.05
*γ*	Total GxE effect	0.05
*ξ*	GxE effect activation curve	10
*υ*	Probability per SNP of GxE	0.1
*β*	Attraction of dynamic environmental factors to phenotype	*U* (0.0175, 0.0325)
*ζ_p_*	Rate of aging as damping of environment and phenotype	$-2\sqrt{\epsilon }$
*ζ_d_*	Rate of aging as damping of environment and phenotype	$-0.2\sqrt{\beta }$
*N*_*g*_	Number of gene SNPs	1000
*N_d_*	Number of dynamic environmental factors	1
*N_e_*	Number of static, additive environmental factors	1000
**Variable**	**Description**	
*g_i_*	SNP effect *i* out of *N*_*g*_ total SNPs	
*P*(*t*)	Phenotypic value at time *t*	
*d_h_*(*t*)	Dynamic environment effect *h* out of *N*_*d*_ total at time *t*	
*l_hi_*	Random nonlinear allelic effect	
*e_j_*	Static, additive environment effect *j* out of *N_e_* environmental factors	
Index	Description	
*h*	Dynamic Environment	
*i*	SNP	
*j*	Static, linear Environment	
*t*	Time	

## Footnotes

1 Arguably, socioeconomic status and many other “environmental” variables also have genetic origins (e.g., Plomin & Bergeman, 1991). For instance, parental genes might impact parental general intelligence which in turn has effects on children’s educational attainment, income, and family socioeconomic status. However, children’s genes do not impact their own socioeconomic status when they are young, although the same genes in the parents can affect family socioeconomic status, a phenomenon called genetic nurture (Kong et al., 2018).

2 Parallel generation of large data sets may not be supported by shinyapps.io. For local model code for data generation, contact the authors.

3 Arguably, the extent to which nonlinearity obfuscates the linear relations may depend on power, particularly the sample size. However, the general pattern that increasing nonlinearity diminishes the ability to find linear relations obtains regardless of power. For example, we repeated the analyses of study 1 with 100,000 people instead of 10,000 people. The results are qualitatively the same: as the nonlinearity increases, the ability to find linear effects is diminished. Instead of finding 26, 4, and 0 Bonferroni-corrected significant SNP effects as reported in the 10,000-person simulation, the 100,000-person simulation finds 62, 40, and 18 SNP effects for the almost linear, moderately nonlinear, and highly nonlinear conditions, respectively. Higher sample sizes find more SNP effects, but higher nonlinearity still impedes finding the effects. The problems introduced by nonlinearity are not solved by increasing the sample size.

4 Because our predictors are simulated SNPs that are in linkage equilibrium and are thus minimally correlated, the *R*^2^ is unlikely to exceed 1. Linkage disequilibrium would imply more strongly correlated SNP predictors and would present a higher chance that *R*^2^ would exceed 1.
